# Cadonilimab in the treatment of advanced metastatic soft tissue clear cell sarcoma achieved complete remission: a case report

**DOI:** 10.3389/fonc.2026.1699788

**Published:** 2026-06-05

**Authors:** Yanzhe Wang, Xiangqin Wang, Mi Meng, Yuju Bai, Hu Ma, Sisi He

**Affiliations:** Department of Oncology, The Second Affiliated Hospital of Zunyi Medical University, Zunyi, China

**Keywords:** advanced metastatic soft tissue clear cell sarcoma, cadonilimab, case report, complete response, PD-1/CTLA-4 bispecific

## Abstract

Soft tissue sarcoma is highly heterogeneous, and clear cell sarcoma (CCS) is a rare, aggressive subtype prone to recurrence and lymph node or lung metastasis. This report describes a patient with upper-limb CCS who developed postoperative recurrence and pulmonary metastasis. After transient benefit from first-line doxorubicin plus ifosfamide chemotherapy, the disease progressed despite second-line PD-1 inhibitor combined with anlotinib. Treatment was subsequently switched to cadonilimab, a PD-1/CTLA-4 bispecific antibody, in combination therapy, resulting in a progression-free survival of more than 24 months. This outcome appears encouraging compared with previously reported CCS benchmarks; however, given the single-case nature and combined treatment strategy, the specific contribution of cadonilimab should be interpreted with caution.

## 1 Introduction

Soft tissue sarcoma (STS) is a group of malignant tumors originating from mesenchymal tissues. It is highly heterogeneous, characterized by local invasiveness and infiltrative growth, with a tendency to recur locally and metastasize to distant sites. The limbs are the most common site of occurrence, followed by the retroperitoneum, trunk, and head and neck. Based on their tissue origin and biological behavior, STS are classified into 12 major categories and over 50 subtypes (1, 2). Clear cell sarcoma (CCS), a rare deep soft tissue malignancy, accounts for about 1% of all soft tissue sarcomas (3). This condition is more prevalent in young patients, primarily affecting the deep fascia of the limbs, followed by the trunk (4). Morphologically and immunophenotypically, CCS resembles malignant melanoma (5). However, it is genetically distinct, most commonly characterized by the EWSR1–ATF1 fusion resulting from t (12;22) translocation (6). CCS is highly aggressive, often leading to regional lymph node and lung metastasis. Approximately one-third of patients present with locally advanced or distant metastasis, resulting in a poor prognosis, with a 5-year survival rate of about 50% (7, 8). The standard treatment for CCS includes surgery, chemotherapy, radiotherapy, immunotherapy, and targeted therapy (9). Surgery is the preferred treatment for early-stage patients, but due to the strong invasiveness of CCS, about half of the patients will experience recurrence after surgery (7), making postoperative adjuvant therapy crucial.

This article presents a case of a rare patient with an upper limb clear cell sarcoma who experienced recurrence and lung metastasis after the initial surgery. After receiving the standard first-line doxorubicin and ifosfamide (AI) chemotherapy regimen, the patient’s condition showed a brief improvement. However, as the disease progressed, the patient received second-line treatment, which included a combination of PD-1 inhibitors and anlotinib for anti-angiogenesis. Despite these treatments, the patient’s condition continued to deteriorate. Given disease progression, an alternative therapeutic strategy was initiated using cadonilimab, a PD-1/CTLA-4 bispecific antibody. Encouragingly, the patient achieved a progression-free survival (PFS) of over 24 months after receiving this treatment. Compared with published CCS-specific benchmarks, including a median PFS of 11 weeks with first-line palliative chemotherapy, 2–4 months in a molecularly confirmed international advanced CCS series, 11 months in the CCS subgroup treated with anlotinib, and a 24-month progression-free rate of 7.7% in the CREATE crizotinib trial, the >24-month PFS observed in this patient appears encouraging. However, this comparison is descriptive and should be interpreted cautiously because this is a single case treated with combination therapy, making it difficult to isolate the contribution of cadonilimab from anlotinib.

## 2 Case report

A 33-year-old male patient visited the Orthopedic Department of Zunyi Medical University Affiliated Hospital in July 2020 due to a ‘‘protrusion on the left forearm.’’ A CT revealed a soft tissue mass in the proximal part of the left forearm, and no distant metastasis was found after comprehensive examinations (**Figures 1A, B**). On July 21, 2020, under general anesthesia, the patient underwent a ‘‘left ulnar and radius proximal tumor resection + resection and reimplantation of the ulna and radius + left thigh iliac muscle bundle resection + left elbow joint ligament repair and reconstruction + left ulna and radius plate and screw internal fixation + left upper limb plaster external fixation.’’ Postoperative pathology indicated a spindle cell tumor in the left forearm. The tumor cells were short spindle-shaped with round nuclei that were deeply stained, with prominent nucleoli and frequent mitosis. Immunohistochemical analysis showed CD34 (-), CD68 (PGM1) (-), CK (-), Desmin (-), EMA (-), Ki-67 (5%), MyoD1 (-), S100 (-), SMA (-), and TFE3 (-) (**Figures 2A, B**). Although S100 negativity is atypical for clear cell sarcoma (CCS), the diagnosis was established based on postoperative pathological findings in combination with clinical features. The tumor was considered to be an intermediate or low-grade malignant mesenchymal tumor. After discharge, the patient declined chemotherapy.

**Figure 1:**
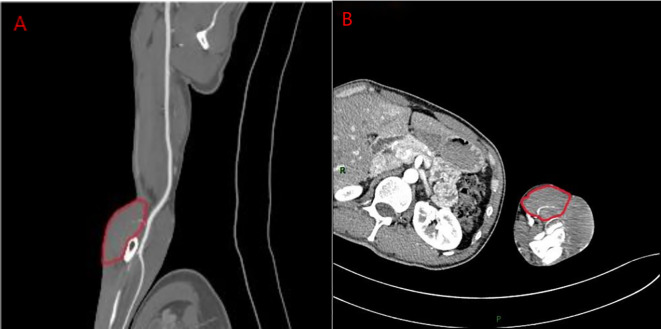
Computed tomography findings of the primary lesion. **(A)** Coronal CT image showing a soft tissue mass in the proximal left forearm. **(B)** Axial CT image showing a soft tissue mass in the proximal left forearm.

**Figure 2:**
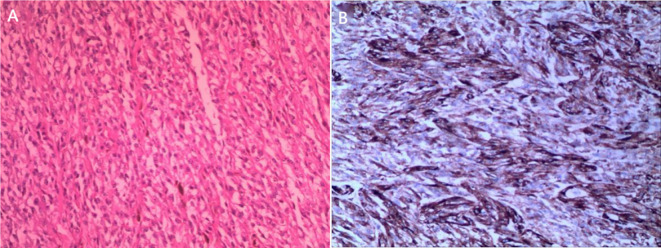
Histopathological features of the tumor. **(A)** Tumor cells were epithelial-like to semi-spindle in shape and arranged in sheets or nests, separated by delicate fibrous connective tissue, with clear cytoplasm. **(B)** Hematoxylin and eosin (H&E) staining showed tumor cells in a pseudopapillary arrangement (original magnification, ×200).

In March 2021, recurrence was detected in the left forearm (**Figures 3A, B**). Further evaluation revealed multiple metastatic lymph nodes in the left axilla (**Figures 3C, D**), multiple bilateral pulmonary metastases (**Figures 3E, F**). Histopathological examination confirmed lymph node metastasis (**Figures 4A, B**). On March 25, 2021, the patient received one cycle of AI chemotherapy regimen, consisting of doxorubicin 90 mg on day 1 and ifosfamide 4 g on days 1–3. After chemotherapy, the patient reported marked pain relief in the left upper limb mass, and the treatment response was evaluated as partial response (PR) (**Figures 5C, D**). However, the patient did not return for the recommended subsequent chemotherapy.

**Figure 3:**
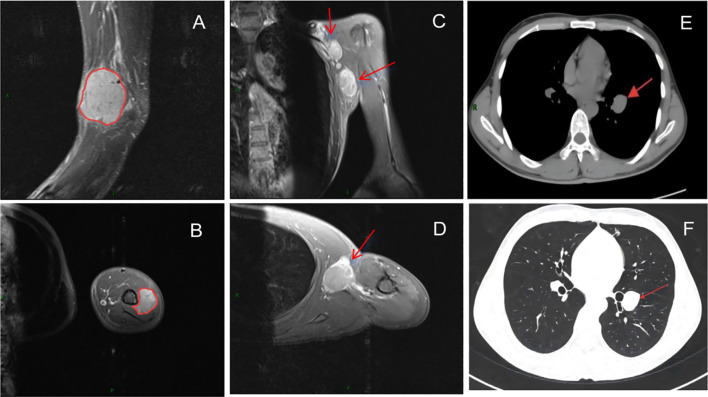
Imaging findings of local recurrence and distant metastases. **(A, B)** MRI images demonstrated recurrent lesions in the left cubital fossa and triceps muscle [**(A)**, coronal view; **(B)**, axial view]. **(C, D)** MRI images demonstrated multiple metastatic lymph nodes in the left axilla [**(C)**, coronal view; **(D)**, axial view]. **(E, F)** Chest CT images demonstrated multiple bilateral pulmonary metastases [**(E)**, mediastinal window; **(F)**, lung window].

**Figure 4:**
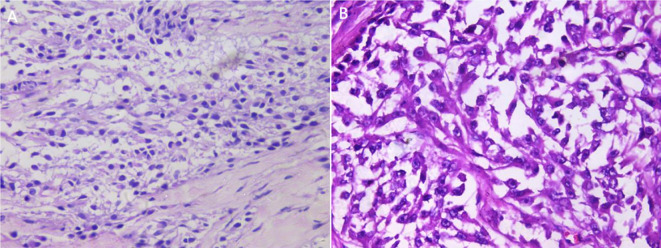
Representative H&E-stained sections of metastatic lymph nodes. **(A)** H&E staining showed tumor cells that were round, oval, or polygonal in shape, with translucent to pale eosinophilic cytoplasm (original magnification, ×200). **(B)** H&E staining showed tumor cells with slightly eccentric nuclei and prominent eosinophilic nucleoli (original magnification, ×400).

**Figure 5:**
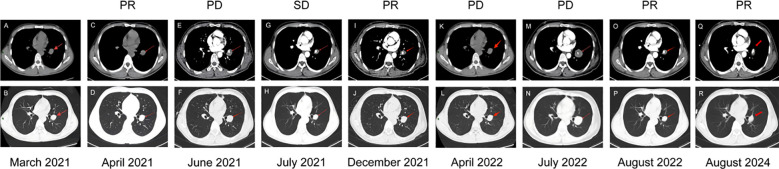
Timeline of systemic treatment and radiological response assessment. Representative chest CT images are shown at different stages of disease progression and treatment. For each time point, paired images show representative target pulmonary lesions; red arrows indicate tumor lesions. **(A, B)** March 2021: baseline imaging showing multiple bilateral pulmonary metastases before systemic therapy. **(C, D)** April 2021: partial response after one cycle of doxorubicin plus ifosfamide chemotherapy. **(E, F)** June 2021: progressive disease after toripalimab combined with anlotinib. **(G, H)** July 2021: stable disease after pembrolizumab combined with anlotinib. **(I, J)** December 2021: partial response after additional AI chemotherapy. **(K, L)** April 2022: progressive disease after postoperative AI chemotherapy. **(M, N)** July 2022: progressive disease before initiation of cadonilimab plus anlotinib. **(O, P)** August 2022: partial response after treatment with cadonilimab plus anlotinib. **(Q, R)** August 2024: follow-up imaging approximately 24 months after initiation of cadonilimab plus anlotinib, showing stable disease without new lesions. Treatment response was assessed according to RECIST criteria. PR, partial response; SD, stable disease; PD, progressive disease; AI, doxorubicin plus ifosfamide.

On April 26 and May 24, 2021, the patient received two cycles of toripalimab combined with anlotinib. Response evaluation showed progressive disease (PD) (**Figures 5E, F**). Subsequently, the treatment regimen was switched to pembrolizumab combined with anlotinib for two cycles, and the response was assessed as stable disease (SD) (**Figures 5G, H**). From August to October 2021, the patient received two additional cycles of AI chemotherapy regimen, during which the mass in the left elbow region decreased significantly, and the response was evaluated as PR (**Figures 5I, J**).

In November 2021, the patient experienced significant bleeding from the left elbow lesion. On November 4, 2021, amputation and axillary lymph node dissection were performed. Postoperative histopathological examination confirmed soft tissue clear cell sarcoma. Between December 1, 2021, and February 10, 2022, the patient received three cycles of AI chemotherapy regimen. However, follow-up chest CT on April 7, 2022, revealed PD (**Figures 5K, L**). Subsequently, the patient underwent two cycles of AI chemotherapy regimen combined with anlotinib, but did not return for further follow-up or treatment thereafter.

In July 2022, the patient developed chest tightness, chest pain, shortness of breath, and worsening cough, and follow-up chest CT again demonstrated PD (**Figures 5M, N**). On August 10, 2022, treatment with anlotinib plus cadonilimab was initiated. Subsequent CT evaluation showed PR (**Figures 5O, P**). Follow-up imaging at approximately 24 months after treatment initiation confirmed sustained disease control (**Figures 5Q, R**).

## 3 Discussion

CCS is a rare and highly aggressive malignant tumor that is generally resistant to traditional chemotherapy and radiotherapy, resulting in limited treatment options and poor prognosis (9). However, with deeper research into the pathogenesis of CCS, targeted therapy and immunotherapy have emerged as promising new treatment strategies, although their efficacy in CCS remains to be fully established (10).

Although immunotherapy has demonstrated activity in certain subtypes of soft tissue sarcoma, its role in CCS remains uncertain. Previous studies of PD-1/PD-L1 inhibitors in sarcomas have reported heterogeneous responses, with generally modest overall response rates (11, 12). For example, in the SARC028 phase II trial, pembrolizumab demonstrated an overall response rate of approximately 18% in selected soft tissue sarcoma subtypes (11), whereas the Alliance A091401 study reported an overall response rate of only about 5% with nivolumab monotherapy (12). These findings highlight the limited and subtype-dependent efficacy of immune checkpoint inhibitors in sarcomas. CCS has often been considered an “immune-cold” tumor, characterized by low tumor mutational burden and limited immune cell infiltration, which may further contribute to the limited efficacy of single-agent immunotherapy (13). Moreover, CCS-specific data are scarce, as most clinical trials include only a small number of CCS patients or do not report outcomes separately (14).

Despite these limitations, there is a biological rationale supporting the potential role of immunotherapy in CCS. CCS is characterized by specific chromosomal translocations such as EWSR1–ATF1, which may generate tumor-associated neoantigens and provide a basis for immune recognition. In addition, anti-angiogenic therapy has been shown to modulate the tumor microenvironment, enhance immune cell infiltration, and potentially improve the efficacy of immune checkpoint blockade (15). Dual immune checkpoint inhibition targeting both PD-1 and CTLA-4 may further enhance antitumor immunity by promoting T-cell priming and activation, thereby overcoming resistance observed with monotherapy.

Cadonilimab is a symmetric tetravalent PD-1/CTLA-4 bispecific antibody with two binding arms for PD-1 and two binding arms for CTLA-4. This structural design allows simultaneous blockade of the PD-1/PD-L1 and CTLA-4/B7 signaling pathways and may enhance antitumor immunity by restoring effector T-cell activity while reducing CTLA-4-mediated T-cell suppression. Compared with the conventional combination of separate anti-PD-1 and anti-CTLA-4 monoclonal antibodies, cadonilimab has several potential mechanistic advantages. First, its tetravalent configuration confers high-avidity binding in settings where PD-1 and CTLA-4 are densely co-expressed, such as the tumor microenvironment, which may promote preferential drug retention and activity at tumor sites. Second, cadonilimab can bind PD-1 and CTLA-4 either on the same T cell through cis-binding or across the T-cell/antigen-presenting cell interface through trans-binding, thereby facilitating local receptor clustering and more efficient immune activation. Third, its Fc-null design minimizes Fc receptor-mediated effector functions, including antibody-dependent cellular cytotoxicity, antibody-dependent cellular phagocytosis, complement-dependent cytotoxicity, and pro-inflammatory cytokine release, which may reduce off-target immune activation and systemic immune-related adverse events. Preclinical studies have shown that cadonilimab can achieve T-cell activation comparable to combined PD-1 and CTLA-4 antibody blockade, while its tumor-selective avidity and Fc-silenced structure may contribute to an improved safety profile. Nevertheless, these mechanistic advantages remain largely supported by preclinical and early-phase clinical evidence, and direct randomized comparisons with conventional PD-1 plus CTLA-4 antibody combinations are still lacking (16, 17). Clinical studies have demonstrated that cadonilimab has a significant synergistic anti-tumor effect on various malignant tumors and shows good safety (18). At the 2023 American Society of Clinical Oncology (ASCO) annual meeting, data from a 2-year follow-up study of cadonilimab combined with chemotherapy as a first-line treatment for gastric/gastric-esophageal junction (G/GEJ) adenocarcinoma in stages Ib/II showed significant efficacy differences in patients with low or negative PD-L1 expression (19). Additionally, Cadonilimab has shown good anti-tumor activity in patients with recurrent or metastatic cervical cancer (20).

This case involves a patient with an upper limb clear cell sarcoma who experienced recurrence after the initial surgery, accompanied by metastasis to lymph nodes and lungs. Following the first cycle of chemotherapy, the patient’s tumor showed signs of remission. However, due to personal reasons, chemotherapy was interrupted. We then selected a second-line treatment regimen, combining a PD-1 inhibitor with anlotinib. Despite continued treatment with the PD-1 inhibitor, the patient’s condition only showed a brief remission after three more cycles of AI chemotherapy regimen, followed by a recurrence. After trying the AI chemotherapy regimen combined with anlotinib, the patient’s condition continued to deteriorate, leading to local recurrence and widespread lung metastasis. Faced with the lack of standard treatment options for third-line therapy, we decided to explore new treatment strategies.

Based on the anti-tumor theory of cadonilimab and the demonstrated anti-tumor activity from related clinical studies, we selected an innovative dual immunotherapy regimen, combining anlotinib anti-angiogenic therapy with cadonilimab, after obtaining informed consent from patients. This innovative treatment approach has overcome the limitations of traditional treatments, achieving a 2-year PFS and demonstrating promising clinical outcomes. Published CCS-specific benchmarks further contextualize this outcome. In a retrospective series of 24 patients with CCS receiving first-line palliative chemotherapy, Jones et al. reported a partial response rate of only 4% and a median PFS of 11 weeks (21). In the largest molecularly confirmed international series of advanced CCS from the World Sarcoma Network, the median PFS across systemic therapies was 2 months, and the most favorable agent-specific result was observed with sunitinib, with a median PFS of 4 months (22). Anti-angiogenic therapy may have activity in CCS; in a phase II study of anlotinib for refractory metastatic soft-tissue sarcoma, the small CCS subgroup had a median PFS of 11 months (23). In the prospective CREATE trial of crizotinib in advanced MET-positive CCS, the median PFS was 131 days and the 24-month progression-free rate was 7.7% (24). Against these CCS-specific benchmarks, the >24-month PFS observed in the present case is notable. Nevertheless, this comparison is descriptive and hypothesis-generating. This report involves a single patient, lacks a control group, and used cadonilimab in combination with anlotinib; therefore, the relative contribution of cadonilimab cannot be separated from anti-angiogenic therapy. In addition, the absence of molecular confirmation, such as EWSR1 rearrangement testing, remains an important limitation.

Given that soft tissue sarcoma is a rare disease, conducting large-scale clinical trials is challenging, leading to treatment plans often being based on limited evidence. In the treatment of clear cell sarcoma, although there is currently a lack of clinical data directly using cadonilimab for CCS treatment, its successful application in other solid tumors and the potential of immunotherapy in CCS treatment suggest that cadonilimab could become a new treatment option for CCS patients. Future clinical studies on the efficacy and safety of cadonilimab in CCS treatment will be crucial. These studies are expected to not only provide more effective treatment options for CCS patients but also further advance the development of tumor immunotherapy, particularly in rare tumor types, offering more treatment possibilities for patients.

In the present case, the patient experienced a durable clinical response following treatment with cadonilimab combined with anlotinib after failure of multiple prior therapies. While this outcome is encouraging, it should be interpreted with caution given the single-case nature and the potential contribution of anti-angiogenic therapy. The absence of molecular confirmation (e.g., EWSR1 rearrangement) represents a limitation of this report and should be considered when interpreting the findings. To our knowledge, this is one of the few reports describing a durable response to a PD-1/CTLA-4 bispecific antibody in CCS. Further prospective studies are required to validate these findings.

## 4 Conclusion

In summary, patients with soft tissue clear cell sarcoma achieved complete remission after receiving PD-1/CTLA-4 bispecific immunotherapy combined with anlotinib targeted therapy. This case provides preliminary evidence that this treatment approach may offer clinical benefit in advanced clear cell sarcoma. Nevertheless, the findings should be interpreted cautiously due to the limitations of a single case report, and further prospective studies are warranted.

## Data Availability

The datasets presented in this study can be found in online repositories. The names of the repository/repositories and accession number(s) can be found in the article/supplementary material.
